# Synergistic effect of acetyl xylan esterase from *Talaromyces leycettanus* JCM12802 and xylanase from *Neocallimastix patriciarum* achieved by introducing carbohydrate-binding module-1

**DOI:** 10.1186/s13568-019-0740-6

**Published:** 2019-01-29

**Authors:** Yueqi Zhang, Hong Yang, Xinrui Yu, Haiyang Kong, Jiaming Chen, Huiying Luo, Yingguo Bai, Bin Yao

**Affiliations:** Key Laboratory for Feed Biotechnology of the Ministry of Agriculture, Feed Research Institute, Chinese Academy of Agricultural Sciences, No. 12 Zhongguancun South Street, Beijing, 100081 People’s Republic of China

**Keywords:** Acetyl xylan esterase, Xylanase, Synergistic effect, CBM1 domain, Xylooligosaccharides

## Abstract

Wheat bran is an effective raw material for preparation xylooligosaccharides; however, current research mainly focuses on alkali extraction and enzymatic hydrolysis methods. Since ester bonds are destroyed during the alkali extraction process, xylanase and arabinofuranosidase are mainly used to hydrolyze xylooligosaccharides. However, alkali extraction costs are very high, and the method also causes pollution. Therefore, this study focuses on elucidating a method to efficiently and directly degrade destarched wheat bran. First, an acidic acetyl xylan esterase (AXE) containing a carbohydrate-binding module-1 (CBM1) domain was cloned from *Talaromyces leycettanus* JCM12802 and successfully expressed in *Pichia pastoris.* Characterization showed that the full-length acetyl xylan esterase AXE + CBM1 was similar toe uncovered AXE with an optimum temperature and pH of 55 °C and 6.5, respectively. Testing the acetyl xylan esterase and xylanase derived from *Neocallimastix patriciarum* in a starch-free wheat bran cooperative experiment revealed that AXE + CBM1 and AXE produced 29% and 16% reducing sugars respectively, compared to when only *NP*XYN11 was used. In addition, introduced the CBM1 domain into *NP*XYN11, and the results indicated that the CBM1 domain showed little effect on *NP*XYN11 properties. Finally, the systematically synergistic effects between acetyl xylan esterase and xylanase with/without the CBM1 domain demonstrated that the combined ratio of AXE + CBM1 coming in first and *NP*XYN11 + CBM1 s increased reducing sugars by almost 35% with AXE and *NP*XYN11. Furthermore, each component’s proportion remained the same with respect to xylooligosaccharides, with the largest proportion (86%) containing of 49% xylobiose and 37% xylotriose.

## Introduction

Plant cell wall is mainly composed of cellulose, hemicellulose and lignin (Bastawde 1992). Xylan is an important component of hemicellulose in plant cell wall and makes up approximately 35% of the dry weight of the cells (Chakdar et al. 2016). It is the second most renewable organic carbon source in nature next to cellulose. Xylan is a kind of complex polysaccharide, and its main chain is a plurality of pyran xylose residues linked by *β*-d-1,4 glycosidic bonds (Biely et al. 1997). However, xylan is a highly polymerized polysaccharide with a wide range of structural changes and branched heteropolysaccharides (Peralta et al. 2017). Different sources of xylan, yield different degrees of branching. Its side chains usually have different substitution groups, such as ferulic acid, *O*-acetyl, coumaric acid, and pyran glucuronic acid (Biely et al. 1985). Acylated xylan is common in hardwoods, and the degree of acylated xylan C-2 and C-3 in the main chain can reach between 50 and 70%, while most of the hydroxyl groups of xylose residues are esterified by acetic acid. Therefore, in the process of biodegradation of xylan, the degradation of acetyl groups with high contents of substituents is particularly important (Collins et al. 2005). Acetyl xylan esterase hydrolyzes the *O*-acetylated glycosaminoglycan on the C-2 and/or C-3 residues of acylated xylan (Biely et al. 1985). The acetyl group interferes with the access of the main chain enzyme in terms of space; therefore, acetyl group removal may enhance the affinity between the enzyme and xylan, thus promoting the effect of xylanase.

Xylooligosaccharides are composed of 2–7 xylose subunits linked by β-1,4 glycosidic bonds. Compared to other oligosaccharides, xylooligosaccharides have many unique functions. They have good acid stability compared to oligofructose, and are extremely resistant to high temperatures (Chen et al. 2009). They also have a strong function in the proliferation of intestinal probiotics, including the highly selective proliferation effect on bifidobacteria, lactic acid bacteria, and other probiotics. In vivo experiments in rats showed that xylooligosaccharides could not only increase the proliferation of probiotics, but also prolong their survival time. In addition, in vitro experiments showed that all bifidobacteria could utilize xylo-disaccharides and xylo-trisaccharides. And these molecules improved ecological balance of microorganisms in animal intestines, producing organic acids and other beneficial substances and reducing the production of ammonia and other decay substances in feces, preventing environmental pollution (Yang et al. 2018). Experiments by Reddy SS and Krishnan C also proved that xylooligosaccharides improved the metabolism and enhanced the humoral immunity of poultry (Reddy and Krishnan 2016).

Currently, the raw materials used to prepare xylooligosaccharides include wheat bran and corncob. Wheat bran accounts for 20% of wheat processing output as a by-product that is of low economic value. It contains approximately 40% xylan which can be divided into water-soluble and water-insoluble xylan based on solubility, with the former constituting approximately 6% of the xylan content in the bran and the xylan being insoluble (Maes and Delcour 2002). Wheat bran xylan and other components of the cell wall (such as lignin and cellulose) are interconnected, and most can be extracted only in alkali solution, as the amount of xylan obtained in direct water extraction is very small. The commonly used methods for xylooligosaccharide extraction involve physical methods, such as hot water extraction, and steam blasting and chemical methods, such as acid and alkali extractions (Rastall 2010). When an ester bond encounters a strong base, it breaks itself. Therefore, many studies focused on the synergistic reaction between xylanase and α-l-arabinofuranosidase in production; however, little work has been done on the synergistic effect of acetyl xylan esterase and xylanase. Alkali extractions of xylooligosaccharides have high recovery efficiency, however, due to the chemical extractions process, consumes considerable amounts of acid, alkali, and ethanol, which pollutes the environment. Since biological methods are relatively safe, the introduction of acetyl xylan esterase and xylanase together is of particular interest. The present study aims to introduce the synergistic effect of xylanase and acetyl xylan esterase on insoluble substrate, as well as analyze of the products for xylooligosaccharides. After acetyl xylan esterase was first discovered by Biely in 1985 (Biely et al. 1985), different types of enzymes have been increasingly discovered and studied. The study by Selig showed that xylanase and acetyl xylan esterase exhibited synergistic effects on the hydrolysis of acetyl xylano (Selig et al. 2009). Puls found that the addition of xylanase and acetyl xylan esterase in different orders not only affected the degree of hydrolysis, but also affected the composition of the hydrolyzed products (Puls et al. 1991). Another study found that adding acetyl xylan esterase can improve the solubility and degradation speed of xylan (Cybinski et al. 1999). In case of an acetyl xylan esterase deficiency, it is difficult for xylanase to move close to the main chain of the highly acetylated xylan; due to reduced accessibility; however, application of acetyl ester enzymes enhance the affinity of xylanase for xylan (Mcdermid et al. 1990).

The xylooligosaccharides enzymatic production method is safe and pollution-free, and the hydrolyzed bran can be further recycled as a favorable feed material (Rastall 2010). However, the recovery rate achieved using this method needs to be improved. A previous study showed that carbohydrate-binding module-1 (CBM1) domain helped GH10 xylanase in hydrolyzing washed corncob particles (Miao et al. 2017). Moreover, Cel5A-CBM6 transgenic plants were 33% more efficient than Cel5A transgenic plants in directly transforming native tobacco cellulose into free sugars (Mahadevan et al. 2011). In this study, the acetyl xylan esterase gene cloned from *Talaromyces leycettanus* JCM12802 was expressed in a *Pichia pastoris* system, and the CBM1 domain was introduced into the xylanase gene from *Neocallimastix patriciarum* to investigate their synergistic effects on starch-free wheat bran.

## Materials and methods

### Strains, vectors, plasmids, kits and culture conditions

The vector pPIC9 was used for construction of the gene, and *Pichia pastoris* GS115 (Invitrogen, Carlsbad, CA, USA) was used for host protein expression. *Escherichia coli* Fast1-T1 (Vazyme, Nanjing, China) receptive cells were used for DNA cloning. T4 DNA ligase purchased from New England BioLabs (Hitchin, UK), was used to connect the genes and vector. Recombinant *Pichia pastoris* was collected from minimal dextrose solid medium (2% glucose, 2% agarose, 1.34% YNB and 4 × 10^−5^ % biotin) and placed into YPD medium (1% yeast extract, 2% peptone, 2% glucose) for 2 days, then saved in an equal volume of YPD and 40% (weight/volume, W/V) glycerol at − 80 °C. Recombinant *P. pastoris* AXEs and *NP*XYN11 s were enriched in BMGY medium (1% yeast extract, 2% peptone, 1% glycerol, 1.34% YNB and 4 × 10^−4^ % biotin) for 2 days at 30 °C with shaking at 200 rpm, then the yeast harvested by centrifugation at 4500 rpm for 7 min and transferred to BMMY medium (1% yeast extract, 2% peptone, 1.34% YNB and 4 × 10^−4^ % biotin) for 2 days with 0.5% methyl alcohol added every 24 h.

### Cloning of genes and sequence analysis of AXE + CBM1 and *NP*XYN11

The SV Total RNA Isolation System (Promega, Tokyo, Japan) was used for extracting the total RNA of the strain *Talaromyces leycettanus* JCM12802 with 3-day-old mycelia in accordance with the specifications. The cDNA fragment of acetyl xylan esterase gene *axe *+ *cbm1* (GenBank accession number MK138893) was used for reverse transcription by the TransScript^®^ One-Step gDNA Removal and cDNA Synthesis SuperMix kit (TransGen, Beijing, China). The peculiar primer sets with restriction sites underlined (*axe *+ *cbm1*-F/R) were designed according to the presumptive gene sequence and amplified from the cDNA of the *axe *+ *cbm1* gene (Table 1). In a similar way, the xylanase *npxyn11* gene (GenBank accession number AF123252.1) from *Neocallimastix patriciarum* was synthesized at Biomed (Beijing, China) by polymerase chain reaction (PCR) with the specific primer *npxyn11*-F/R (Table 1). The purified PCR product was connected to the pEASY-T3 vector (TransGen) and transformed thermally at 42 °C for 90 s into *Escherichia coli* Fast1-T1 cells.

**Table 1:** Primers used in the study

Prime name	Sequences (5′ → 3′)
*axe *+ *cbm1*-F	GAATTCGTTGCGGTGGATCACGATG^a^
*axe *+ *cbm1*-R	GCGGCCGCTCACAGACATTGATAATAGTAATCATTGAC^a^
*axe*-F	GAATTCGTTGCGGTGGATCACGATG^a^
*axe*-R	GCGGCCGCTCAAGCAAATCCAAACCATTCC^a^
*cbm1*-F	GGTGAGTCTACTGGTGGCGGAA
*cbm1*-R	TCACAGACATTGATAATAGTAATCATT
*npxyn11*-F	GAATTCCAAAGTTTCTGTAGTTCAGCTTCT^a^
*npxyn11*-R	GCGGCCGCTCAATCACCAATGTA^a^
*npxyn11*gene-F	CAAAGTTTCTGTAGTTCAGCTTCT
*npxyn11*gene-R	ATCACCAATGTAAACCTTTGCGTATG

^a^Nucleotides incorporated for restriction enzyme digestion are underlined. *Eco*RΙ: GAATTC; *Not*Ι: GCGGCCG

### Construction of mutants

Sequence and structural analyses (http://www.ncbi.nlm.nih.gov/BLAST/) showed that the acetyl xylan esterase AXE + CBM1 from strain JCM12802 has a specific sequence of the cellulose-binding module CBM1. Four different protein domains are present in this enzyme: a signal peptide (1–19 amino acids), a hydrolase domain (20–309), a S/T-rich linker region (310–342) and a CBM1 domain (343–380). To explore the specific functions of the CBM1 domain, introduced it to the C-terminal of the xylanase *NP*XYN11. The *cbm1* sequence was removed from the acetyl xylan esterase gene *axe *+ *cbm1* and added to the xylanase gene *npxyn11* using peculiar primer sets with PCR formed the xylanase gene *npxyn11 *+ *cbm1* (GenBank accession number MK138894) (Table 1). The annealing temperature is based on primers in the PCR.

### Expression and purification of AXE + CBM1, *NP*XYN11 and mutants

The recombinant plasmids were linearized by a FastDigest *Bgl*II restriction enzyme (Thermo Scientific, Waltham, MA, USA), then purified using the Gel Extraction Kit (Omega Bio-Tek, Norcross, GA, USA). The plasmid purifications were performed utilizing the Gene Pulser Xcell electroporation system (Bio-Rad, Hercules, CA, USA), then transformed into *P. pastoris* GS115 competent cells. Recombinant *P. pastoris* AXEs and *NP*XYN11 s proteins were obtained by high-speed refrigerated centrifugation (CR21GIII, Hitachi, Japan) in clear liquid at 12,000 rpm for 3 min at 4 °C, and a 10 kDa molecular weight cut off PES (Sartorius, Germany) was used for ultrafiltration and concentration. Before purifying the crude enzymes, needed to desalinate them using 20 mM citric acid-Na_2_HPO_4_ (pH 6.9) for AXE + CBM1 s and 20 mM citric acid-Na_2_HPO_4_ (pH 7.7) for *NP*XYN11 s. The desalted enzymes AXE + CBM1 and *NP*XYN11 were equilibrated with 20 mM citric acid-Na_2_HPO_4_ (pH 6.9) and citric acid-Na_2_HPO_4_ (pH 7.7) and placed into a HiTrap Q Sepharose XL 5-mL fast protein liquid chromatography (FPLC) column (GE Healthcare, Uppsala, Sweden). A flow velocity of 1.5 mL min^−1^ and a linear gradient of NaCl solution (0–1.0 M) were used to elute enzymes. To respectively use the acetyl xylan esterase enzyme activity and xylanase enzyme activity for measuring their biochemical characterization, sodium dodecyl sulfate–polyacrylamide gel electrophoresis (SDS-PAGE) was employed including a 12% separation gel and a 5% stacking gel for observing their protein molecular weight and purity. An Easy Protein Quantitative Kit (TransGen, Beijing, China) was used to detect protein concentration.

### Substrates and enzyme activity assay

All samples were examined in triplicate. The substrate used for xylanase enzyme activity was beechwood xylan (Sigma, St. Louis, USA), and the standard reaction system included placing 0.1 mL appropriately diluted enzyme appropriately and 0.9 mL of 0.5% (w/v) beechwood xylan in 0.1 M citric acid-Na_2_HPO_4_ (pH 6.0) in a 65 °C thermostatic water bath for 10 min. Afterwards, 1.5 mL 3, 5-dinitrosalicylic acid reagent (DNS) was added and a 5-min immersion in boiling water for the sake of termination reaction. The control samples were added to the enzymes after the addition of DNS, and cooled to room temperature, next all samples were examined using spectrophotometer at 540 nm absorbance. Under the conditions assayed in this study, the per-minute quantity released by the enzyme of 1 μmol reducing sugar was defined as one unit of xylanase enzyme activity.

4-Nitrophenyl acetate (*p*NPA, Sigma, St. Louis, USA) was used as the substrate for the acetyl xylan esterase enzyme, and the *p*NPA solution was prepared from 0.09 g *p*NPA dissolved in 5 mL dimethyl sulfoxide (DMSO, Sigma) in this study. The accurate reaction system contained 0.1 mL appropriately diluted enzyme, 0.04 mL *p*NPA solution and 20 mM citric acid-Na_2_HPO_4_ (pH 6.0) placed at 55 °C in a thermostatic water bath for 10 min, after which added to 1 mL absolute ethyl alcohol was added to end the reaction. Under the standard assay conditions, one unit of xylanase enzyme activity was defined as the quantity of 1 μmol of *p*-Nitropheno released by the enzyme per minute. The substrate specificities of acetyl xylan esterase enzyme activity were 4-Nitrophenyl acetate (C2), 4-Nitrophenyl butyrate (C4), 4-Nitrophenyl octanoate (C8), 4-Nitrophenyl decanoate (C10) and 4-Nitrophenyl decanoate (C12) from Sigma. 7-Aminocephalosporanic acid (7-ACA) also purchased from Sigma and prepared in a 1‰ 7-ACA solution with 0.2 M citric acid-Na_2_HPO_4_ (pH 8.0).

### Biochemical characterization and kinetic parameters assay

For the xylanase enzyme activity assay, the buffers used are listed as follows: 100 mM glycine–HCl (pH 2.0), 100 mM citric acid-Na_2_HPO_4_ (pH 3.0–8.0), 100 mM Tris–HCl (pH 8.0–9.0), and 100 mM glycine–NaOH (pH 9.0–12.0). The optimum temperature for xylanase enzymes were determined by measurement at the optimal pH from 45 to 75 °C incremented by 5 °C. By the same token, the optimum pH was found by measurements in the range of 3.0–8.0 at optimal temperature. The pH stability of xylanase was examined by placing the enzymes in a water bath at 37 °C for 1 h in the abovementioned buffer, then assaying appropriately diluted enzyme at the optimal conditions of 60 °C and pH 5.5 for 10 min. The controls used uninsulated enzymes. To determine the temperature stability, samples were cultivated at the optimal pH for 0, 5, 10, 20, 30, and 60 min at 65 °C, 75 °C and 80 °C, then placed into ice. The control sample was the 0 min cultivation into ice, and residual enzyme activities were calculated at optimal conditions. Through different catalytic concentrations of beechwood xylan at 10 mg mL^−1^, 8 mg mL^−1^, 6 mg mL^−1^, 5 mg mL^−1^, 4 mg mL^−1^, 2 mg mL^−1^ and 1 mg mL^−1^, the Lineweaver–Burk equation (x-axis gyroscope as the reciprocal of the substrate concentration, y-axis gyroscope as the reciprocal of the enzyme reaction velocity) was used to calculate the Michaelis–Menten constant (*K*_m_), catalytic rate constant (*k*_cat_) and maximal velocity (*V*_max_) under the optimal conditions in triplicate.

To determine the acetyl xylan esterase enzyme activity, buffers were prepared as shown: 20 mM glycine–HCl (pH 2.0), 20 mM citric acid-Na_2_HPO_4_ (pH 3.0–8.0), 20 mM Tris–HCl (pH 8.0–9.0), and 20 mM glycine–NaOH (pH 9.0–12.0). The optimum temperature for acetyl xylan esterase enzymes was determined by measurements at the optimal pH up 20 °C to 80 °C by 10 °C increments. The optimum pH was then determined by measurements in the range of 3.0–8.0 at the optimal temperature, but 1 mM *p*NPA was used as the substrate for pH 7.0–8.0, and the *p*NPA concentration was 1 mM for the rest of the conditions. The same procedure was used for the optimum temperature measurements in the range from 20 °C to 80 °C per 10 °C under pH 6.0. For pH and temperature stability assessments, the same procedure was used as that implemented for the xylanase enzyme activity assay, only different in that the samples were cultivated at the optimal pH for 0, 5, 10, 20, 30, and 60 min at 55 °C and 65 °C. Similarly used 0.5 mM, 1 mM, 2 mM, 3 mM, 4 mM, 6 mM and 8 mM *p*NPA were used to measure the *K*_m_, *k*_cat_ and *V*_max_ values. The calculation of *k*_cat_/*K*_m_ for the substrate of 7-ACA was determined by consulting the method of Matsui’s research using high performance liquid chromatography (HPLC) (Matsui et al. 1991).

### Fabrication of starch-free wheat bran at collaborative experiment

Approximately 500 g dry wheat bran was weighed, and washed twice in pure water. Then the cleared wheat bran was placed in 2 L pure water in a pot and heated until boiling, then let cool slightly before adding 1 mL thermostable amylase. The above steps were three times. Finally the wheat bran was placed into the stove at 60 °C overnight. Afterward, the wheat bran was processed with a pulverized and sieved through 50 mesh sieve. Concentration of 2%, 5%, 10% starch-free wheat bran were made by weighing 0.4 g, 1 g and 2 g starch-free wheat bran and dissolving in 20 mL 20 mM citric acid-Na_2_HPO_4_ (pH 6.0) in 100 mL triangular flasks.

The xylanase solution was calibrated by units of enzyme activity, diluted and put into different test tubes to achieve 25 U, 50 U, 100 U, 200 U, 400 U, 800 U and 1600 U solutions. The reaction system included 0.4 g starch-free wheat bran, 19 mL pure water and 1 mL *NP*XYN11 at 50 °C for 1 h and 100 rpm. After the reaction, 1 mL of the supernatant of the appropriate dilution ratio was collected, and 1.5 mL DNS was added to detect the reducing sugar content. The addition of xylanase was determined in the early stage by adding different units (0 U, 25 U, 50 U, 100 U and 200 U) of acetyl xyaln esterase AXE + CBM1, and afterwards, increments of reducing sugar content were used to ensure the addition of acetyl xylan esterase. After determining the amount of xylanase and acetyl xylan esterase, the order of addition and the influence of substrate concentration on the synergistic effect were examined.

The hydrolysis products of starch-free wheat bran were obtained by adding 50 U acetyl xylan esterase AXE or AXE + CBM1 first, then 200 U xylanase *NP*XYN11 and *NP*XYN11 + CBM1, followed by incubation at 50 °C for 1 h. For detection of the main hydrolysis products, high-performance anion-exchange chromatography (HPAEC) DIONEX ICS-5000 (Thermo Scientific, Sunnyvale, USA) was used with a CarboPac PA200 analysis column (3 mm × 250 mm) and 1 mol L^−1^ NaOH eluant. The standard substances for the products were xylose (Sigma), xylobiose, xylotriose, xylotetraose, xylopentaose, and xylohexaose from Megazyme, Ireland.

## Result

### Gene cloning and sequence analysis of *axe *+ *cbm1*, *npxyn11* and their mutants

**Fig. 1:**
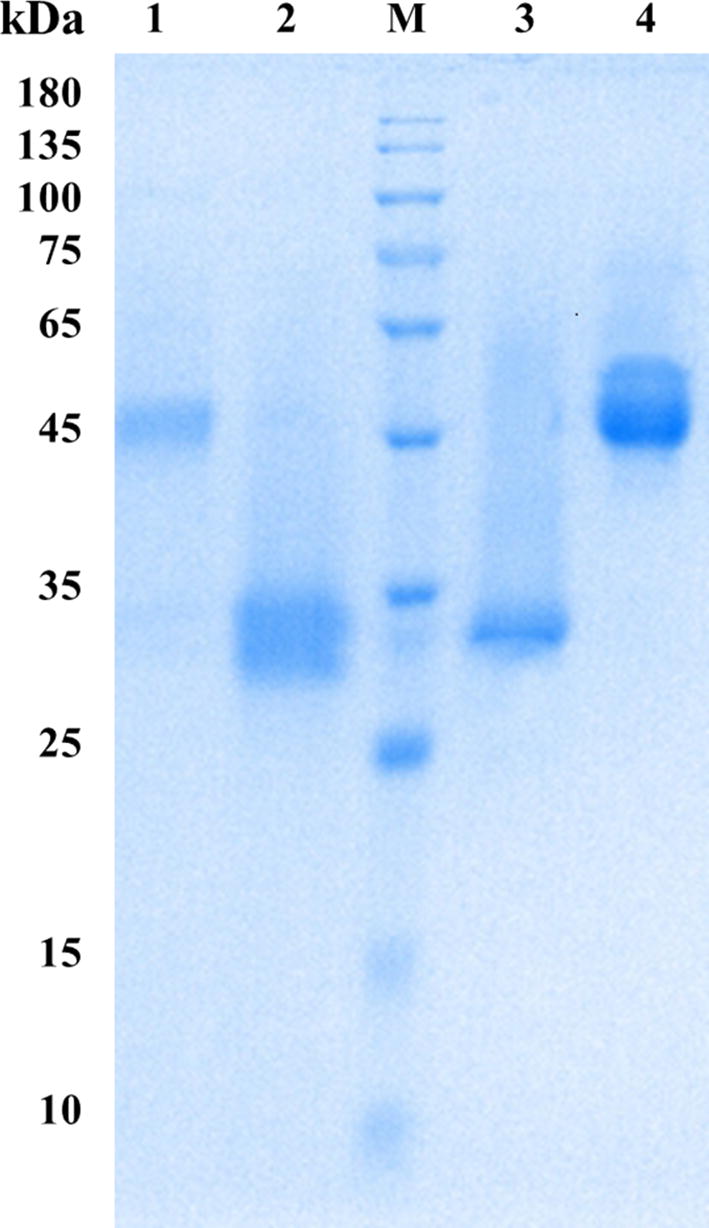
SDS-PAGE analysis of the purified protein *NP*XYN11, *NP*XYN11 + CBM1, AXE and AXE + CBM1. Lane M, the standard protein molecular weight markers; lane 1, the purified AXE + CBM1; lane 2, the purified AXE; lane 3, the purified NPXYN11; lane 4, the purified NPXYN11 + CBM1

The full-length cDNA of the acetyl xylan esterase gene *axe *+ *cbm1* was 1083 bp in length according to the display of SnapGene software and was cloned from the cDNA of JCM12802. The CBM1 domain mainly consists of one linker domain and one mature CBM1 domain. In this study, the meaning of the CBM1 domain includes the linker and the mature CBM1. The full-length CBM1 was 213 bp in length. The mature AXE + CBM1 and AXE protein have an isoelectric point of 4.88, and their molecular were 38.4 kDa and 31.3 kDa, respectively, as obtained from the prediction of Vector NTI software. Purified proteins all displayed the same molecular mass on SDS-PAGE (Fig. 1).

### Basic properties of AXE + CBM1 and its mutant, AXE

**Fig. 2:**
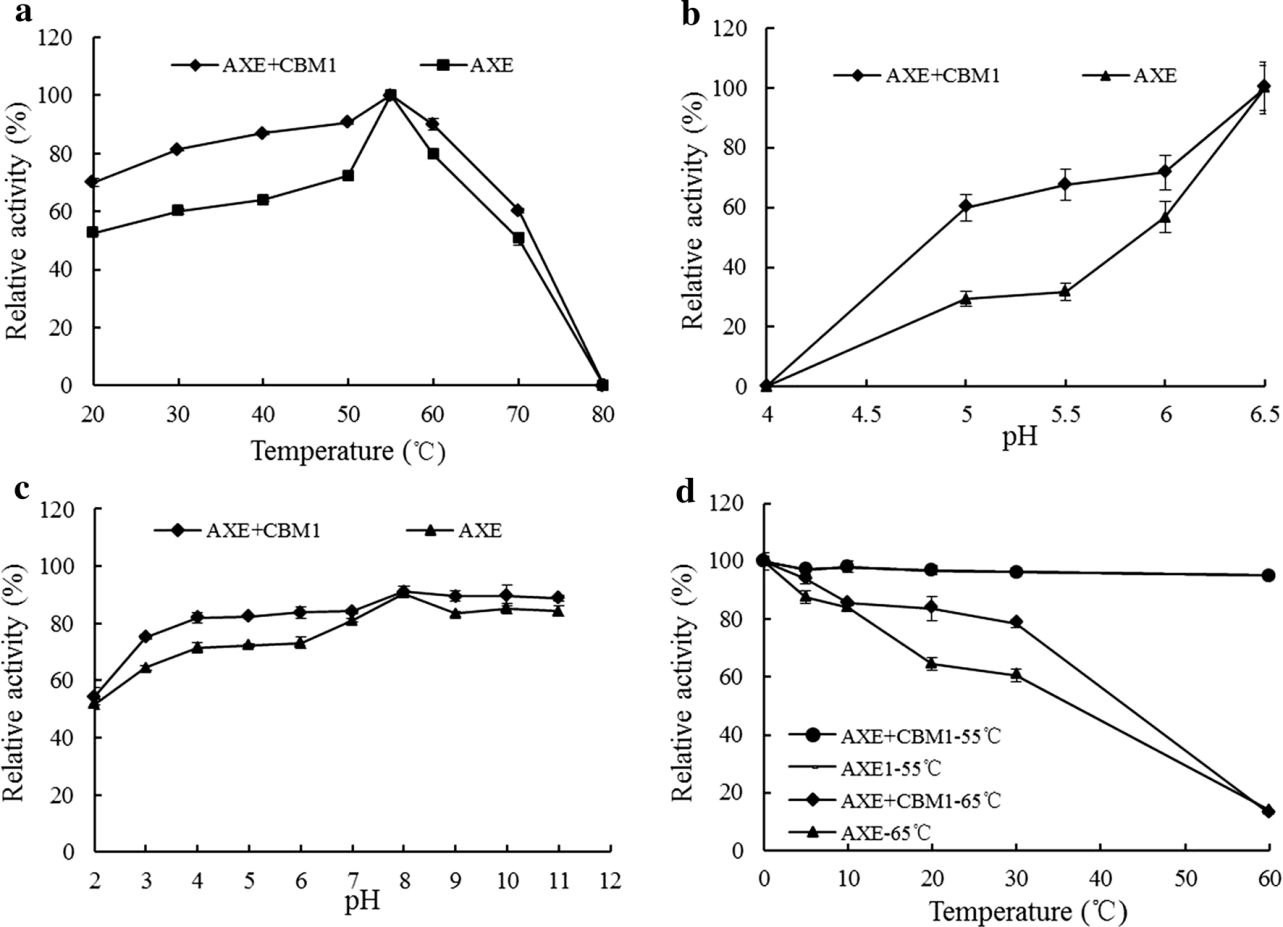
Characterization of the purified AXE and AXE + CBM1. **a** Effect of temperature on the AXE and AXE + CBM1 activities. **b** Effect of pH on the AXE and AXE + CBM1 activities. **c** Stability of pH on the AXE and AXE + CBM1 activities. **d** Thermostability assay on the AXE and AXE + CBM1 activities. Each value in the panel represents the mean ± SD (n = 3)

AXE + CBM1 and its mutant AXE had the optimal temperature of 55 °C, as well as residual 80% enzyme activity for AXE + CBM1 and 60% enzyme activity at the range of 30–60 °C for AXE for the substrate of *p*NPA (Fig. 2a). If the *p*NPA concentration is too high, the reaction solution will increasingly grow yellow in portions, which could not be read in the microplate reader at 410 nm, even if the solution was a blank control at alkaline conditions. Hence, the current experiment selected a 4 mmol concentration of *p*NPA within pH 4.0–6.5. On account of pH stability, AXE + CBM1 and AXE exhibited quite broad range of pH 3.0–11.0 and retained more than 60% of the initial enzyme activity (Fig. 2c). AXE + CBM1 and AXE already showed favorable stability at 55 °C within 1 h, but at 65 °C for 1 h, acetyl xylan esterase enzyme activities were lost completely. However, AXE + CBM1 retained 80% of its enzyme activity and AXE retained 60% under the condition of 65 °C for half an hour (Fig. 2d).

When *p*NPA was used as the substrate, AXE + CBM1 and AXE had light differences in terms of the *K*_m_, *V*_max_ and *k*_cat_ (Table 1). Though AXE showed lower *k*_cat_/*K*_m_ and *K*_m_ values than AXE + CBM1, its *V*_max_ and *k*_cat_ values were higher than did AXE +CBM1 (Table 2). The figures indicate that the CBM1 domain of acetyl xylan esterase was conducive to compatibility between the enzyme and substrate.

**Table 2:** Kinetic values of acetyl xylan esterase and xylanase

Protein	*K*_m_ (mM)	*V*_max_ (μmol min^−1^ mg^−1^)	*k*_*cat*_ (s^−1^)	*k*_cat_/*K*_m_ (s^−1^ mM^−1^)
AXE	0.72 ± 0.03	625 ± 21	326 ± 17	453 ± 18
AXE + CBM1	0.5 ± 0.02	455 ± 19	306 ± 9	612 ± 12

Protein	*K*_m_ (mg mL^−1^)	*V*_max_ (μmol min^−1^ mg^−1^)	*k*_*cat*_ (s^−1^)	*k*_cat_/*K*_m_ (mL s^−1^ mg^−1^)
*NP*XYN11	4.5 ± 0.6	5128 ± 166	2085 ± 26	459 ± 9
*NP*XYN11 + CBM1	5.8 ± 0.7	6536 ± 191	3437 ± 19	584 ± 8

Compared to experiments using *p*NPA, AXE + CBM1 and AXE demonstrated extremely low *k*_cat_/*K*_m_ values with the substrate 7-ACA. The earlier experiment used *p*NPA as the substrate to calibrate the enzyme activities of AXE + CBM1 and AXE. Upon on the addition of identical active units of AXE + CBM1 and AXE and reacting with 1‰ 7-ACA for 1 h, the value of *k*_cat_/*K*_m_ of AXE + CBM1 was slightly larger than AXE, with the value of 17.45 mmol^−1^ min^−1^ and 15.04 mmol^−1^ min^−1^ respectively. This result expressed that the CBM1 domain was related to the catalytic efficiency of AXE + CBM1.

At the optimum conditions of temperature and pH, the efficiency of hydrolysis of acetyl xylan esterase AXE + CBM1 and 4-Nitrophenyl acetate was the highest. In addition, acetyl xylan esterase AXE + CBM1 and 4-Nitrophenyl butyrate (C4) only retained approximately 15% of the enzyme acyivity compared to the sample with the substrates of 4-Nitrophenyl acetate. Nevertheless 4-Nitrophenyl octanoate (C8), 4-Nitrophenyl decanoate (C10), 4-Nitrophenyl decanoate (C12) as the substrate respectively, enzyme activities were not entire detected. In general, acetyl xylan esterase AXE + CBM1 was more in favor of short-chain fatty acids than *p*NP-ester substrate.

### Basic properties of *NP*XYN11 and its mutant *NP*XYN11 + CBM1

**Fig. 3:**
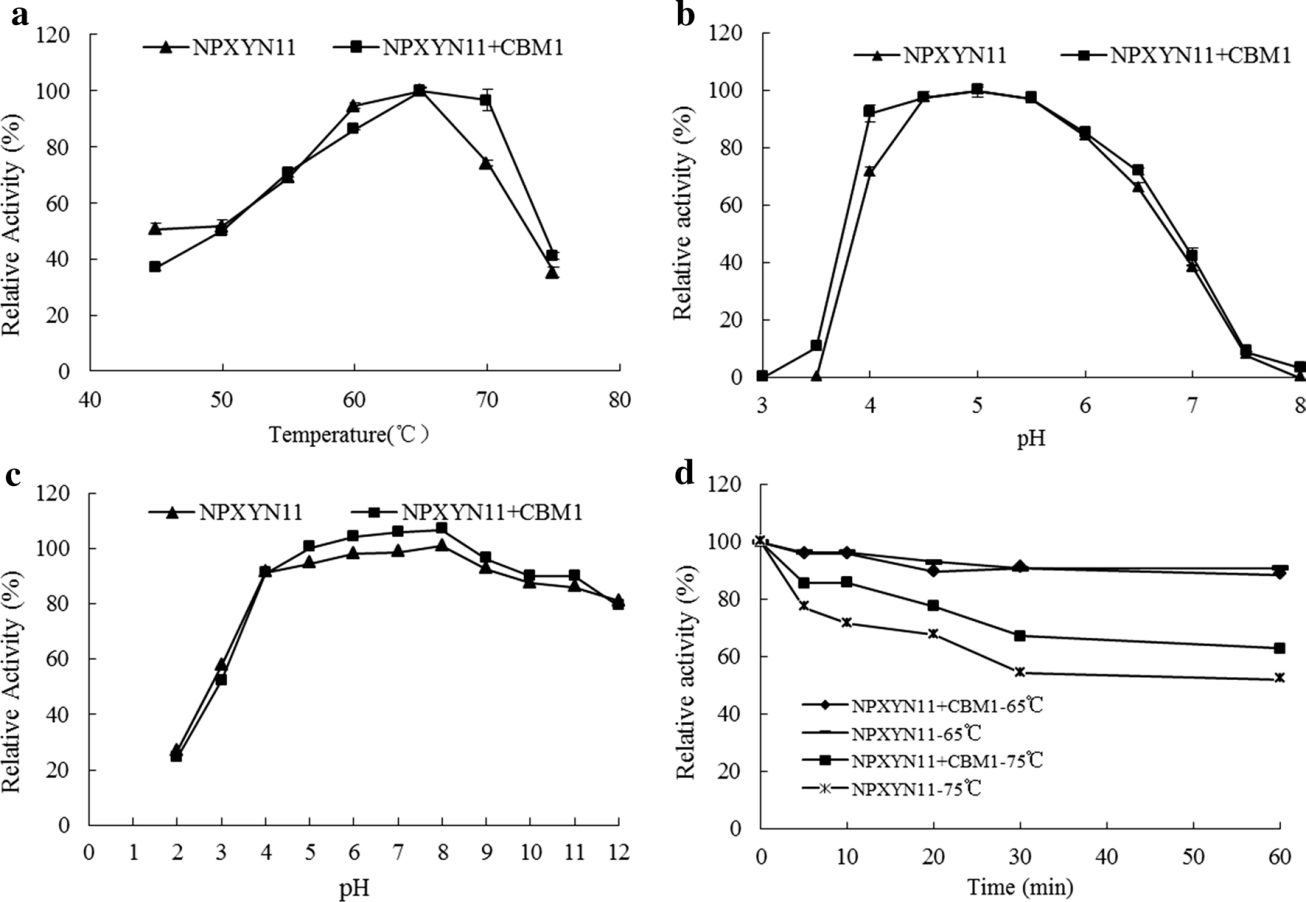
Characterization of the purified *NP*XYN11 and *NP*XYN11 + CBM1. **a** Effect of temperature on the *NP*XYN11 and *NP*XYN11 + CBM1 activities. **b** Effect of pH on the *NP*XYN11 and *NP*XYN11 + CBM1 activities. **c** Stability of pH on the *NP*XYN11 and *NP*XYN11 + CBM1 activities. **d** Thermostability assay on the *NP*XYN11 and *NP*XYN11 + CBM1 activities. Each value in the panel represents the mean ± SD (n = 3)

The optimal temperatures of *NP*XYN11 and its mutant *NP*XYN11 + CBM1 were both 65 °C, and *NP*XYN11 + CBM1 retained 90% of its enzyme activity or more within the range from 60 to 70 °C (Fig. 3a). *NP*XYN11 and *NP*XYN11 + CBM1 retained more than 70% enzyme activities at pH 4.0–6.0, and 5.0 was the optimal pH for both (Fig. 3b). From the diagram results, the thermal stability of *NP*XYN11 + CBM1 was a little better than *NP*XYN11. Within the range of pH 4.0–11.0 for 1 h, *NP*XYN11 and *NP*XYN11 + CBM1 retained more than 80% of their enzyme activities compared with untreated protein. Furthermore they had approximately 100% enzyme activities at pH 5.0–8.0 for 1 h (Fig. 3c). After incubation at 65 °C for 1 h, *NP*XYN11 and *NP*XYN11 + CBM1 were stable and retained 95% enzyme activities. At 75 °C and 80 °C for 1 h, *NP*XYN11 + CBM1 maintained 65% and 43% residual enzyme activities respectively, while *NP*XYN11 had 58% and 40% (Fig. 3d).

When beechwood xylan was used as the substrate for *NP*XYN11 and *NP*XYN11 + CBM1, the values of *V*_max_, *k*_cat_ and *k*_cat_/*K*_m_ all were on the rise (Table 2). From the data in the table, Vmax, *k*_cat_ and *k*_cat_/*K*_m_ of *NP*XYN11 were slightly less than those of *NP*XYN11 + CBM1, indicated that the CBM1 domain was beneficial to the binding of xylanase *NP*XYN11 and substrate.

### The synergistic reaction with xylanase and acetyl xylan esterase

**Fig. 4:**
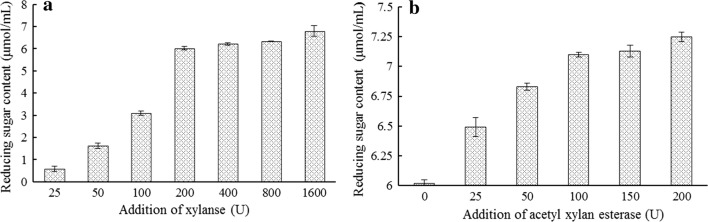
Determined addition of acetyl xylan esterase and xylanase in starch-free wheat bran experiment as reducing sugar. **a** The content of reducing sugar produced by xylanase *NP*XYN11 in different units of activity to the starch-free wheat bran. **b** Reducing sugar content on the basic of 200 U xylanase *NP*XYN11 and different amount acetyl xylan esterase AXE severally

Xylanase solution was calibrated using units of enzyme activity, diluted and put into different test tubes to achieve different enzymatic units from 25 U-1600 U. Basic of the result in the table below, reducing sugar increment was approximately proportional to the amount of addition at the range of 25–200 U (Fig. 4a). When the additive amount of xylanase was 200 U, the yield of reducing sugar was 6 μmol mL^−1^ approximately, and when the addition amount of xylanase was increased exponentially, but the amount of reducing sugars was not doubled. This result was the reason why *NP*XYN11 or *NP*XYN11 + CBM1 were added at 200 U in the collaborative experiment below using acetyl xylan esterase and xylanase.

The addition of xylanase was determined in the early stage by adding different units of acetyl xylan esterase AXE + CBM1. Afterwards the increment of reducing sugar content was used to ensure the addition of acetyl xylan esterase. According to the following result, the reducing sugar increment was approximately proportional to the amount of addition within the range of 25–50 U (Fig. 4b). When the addition of AXE + CBM1 exceeded 50 U, the increment of reducing sugar was less. It was the reason why AXE + CBM1 or AXE was added 50 U in the collaborative experiment of below acetyl xylan esterase and xylanase.

In the early grouping, the addition amounts of acetyl xylan esterase and xylanase were determined and it is found that acetyl xylan esterase was conducive to xylanase activity in the degradation of insoluble substrates. By means of different orders of addition of acetyl xylan esterase and xylanase, single-enzyme and double-enzyme reactions and the adding order were explored for the collaborative experiment. The experimental results show that the most appropriate solution was the addition of xylanase after acetyl xylan esterase, and the reducing sugar content from this method was the maximum (29%) under similar conditions (Table 3).

**Table 3:** Order of addition about acetyl xylan esterase and xylanase on starch-free wheat bran

Order	AXE and *NP*XYN11		AXE + CBM1 and *NP*XYN11	
	Reducing sugar content (μmol mL^−1^)	Percentage of promotion with order 2 (%)	Reducing sugar content (μmol/mL)	Percentage of promotion with order 2 (%)
1^a^	ND		ND	
2^b^	5.97 ± 0.18		5.97 ± 0.18	
3^c^	6.91 ± 0.11	16	7.72 ± 0.21	29
4^d^	6.21 ± 0.17	4	6.33 ± 0.19	6
5^e^	6.51 ± 0.11	9	6.69 ± 0.02	12

^a^Only added 50 U acetyl xylan esterase at 50 °C water bath for 100 rpm at 1 h

^b^Only added 200 U xylanase at 50 °C water bath for 100 rpm at 1 h

^c^Added 50 U acetyl xylan esterase first at 50 °C water bath for 100 rpm at 1 h. Then boiled 5 min for deactivation and added 200 U xylanase next for reaction at 50 °C for 100 rpm at 1 h

^d^Added 200 U xylanase first at 50 °C water bath for 100 rpm at 1 h. Then boiled 5 min for deactivation and added 50 U acetyl xylan esterase next for reaction at 50 °C for 100 rpm at 1 h

^e^Simultaneously added 50 U acetyl xylan esterase and 200 U xylanase for reaction at 50 °C water bath at 1 h for 100 rpm

If other conditions were exactly the same, the higher was the substrate concentration or the longer was the reaction time, the more obvious was the promotion of reducing sugar content (Table 4). Compared with xylanase alone, the addition of double-enzymes had a similar effect (Table 5). In terms of the results, the CBM1 domain of acetyl xylan esterase or xylanase was favorable for producing more reducing sugars. Under the comprehensive consideration, the cooperative experiment used the combination *NP*XYN11 + CBM1 and AXE + CBM1 with combination *NP*XYN11 and AXE to degrade 10% starch-free wheat bran at 50 °C and 100 rpm for 1 h. The result showed that reducing sugar content was ultimately increased 34% when then reaction was performed with the addition of *NP*XYN11 + CBM1 and AXE + CBM1.

**Table 4:** The impact of starch-free wheat bran concentration about acetyl xylan esterase and xylanase

Protein	Starch-free wheat bran concentration (%)	Reducing sugar content at 60 min (μmol mL^−1^)	Percentage of promotion (%)
*NP*XYN11	2	5.56 ± 0.03	
*NP*XYN11	5	8.92 ± 0.11	
*NP*XYN11	10	15.71 ± 0.16	
*NP*XYN11 + CBM1	2	6.03 ± 0.04	8
*NP*XYN11 + CBM1	5	10.19 ± 0.12	14
*NP*XYN11 + CBM1	10	19.01 ± 0.21	21

**Table 5:** The influence of the CBM1 domain with/without acetyl xylan esterase and/or xylanase on starch-free wheat bran

Protein	Starch-free wheat bran concentration (%)	Reducing sugar content (μmol mL^−1^)	Percentage of promotion (%)
AXE and *NP*XYN11	2	6.94 ± 0.03	
AXE and *NP*XYN11	5	10.28 ± 0.09	
AXE and *NP*XYN11	10	17.40 ± 0.04	
AXE + CBM1 and *NP*XYN11 + CBM1	2	7.66 ± 0.1	10
AXE + CBM1 and *NP*XYN11 + CBM1	5	12.27 ± 0.07	19
AXE + CBM1 and *NP*XYN11 + CBM1	10	23.18 ± 0.08	34

## Discussion

Xylooligosaccharides are functional oligosaccharides with unique physiological activities, linked with multiple xylose molecules via β-1,4-glycosidic bonds. Due to their remarkable functional benefits in feed production, xylooligosaccharides have been extensively studied by increasing numbers of scholars. A study showed that day-old, healthy Arbor Acres broilers that were fed a basal diet (maize–soybean meal) containing 10 g kg^−1^ of xylooligosaccharides were observed to gain 9.44% more weight on the 59th day when compared to the control group that did not have xylooligosaccharides in their diet (Sun et al. 2013). Moreover, the avian influenza H5N1 virus incidence was significantly higher (by 33.78%) than in the control group by 33.78% for when compared to the treatment group with that had the same amount of xylooligosaccharides added to their feed. The solubility of the bone mineral bioapatite was also found to be lower and the crystallinity was found to be higher (leading to improved bone mineralization) than in the experimental group after adding 0.1–0.5 g kg^−1^ of xylooligosaccharides during the growing and fattening stage in pigs when compared to that of the control group (Wang et al. 2017). In mice with adenine-induced chronic kidney disease, xylooligosaccharides interfered with the reduction process of six out of nine bacterial genera in chronic kidney diseases (Yang et al. 2018). Xylooligosaccharides also have important roles in increasing intestinal probiotics and improving the intestinal microecological environment (Ho et al. 2014). A study in male Sprague–Dawley rats showed that the serum triglyceride concentrations were reduced by 34% when mice were supplemented with 60 g kg^−1^ xylooligosaccharides in their feed after 35 days, and that *Bifidobacterium* levels increased and *Escherichia coli* levels decreased in cecal microbiota (Hsu et al. 2004). Adding xylooligosaccharides to the feed effectively reduce its feed production coefficient, improved the growth performance of poultry, and improve animal immunity and metabolic functions.

Preparation methods using corncobs, the main raw material for xylooligosaccharide production, mainly include acid–base extraction (Quinones et al. 2015), hot water hydrolysis (Vazquez et al. 2000), steam explosion (Shimizu et al. 1998) and enzymatic hydrolysis (Strelova and Chuvina 2008). However, the high costs involved in alkali extraction (Qi et al. 2009) and the need for a series of complex processes, such as ethanol dissolution, precipitation, desalination and decolorization limit its efficient utilization. For example, xylanase is used to decompose the xylan backbone, and coenzymes such as acetyl xylan esterase and α-l-arabinofuranosidase are used to degrade side chains in the enzymatic degradation of xylan from corn cobs. However, the enzymatic hydrolysis of corn husks and corn cobs is extremely inefficient. Surprisingly, direct hydrolysis of wheat bran and corn husks by xylanase resulted in 7–8 times (data not shown) more product from the wheat bran reducing sugar content when compared to the same in the corn husk. Therefore, one-step enzymatic hydrolysis provides a possibility for producing xylooligosaccharides. Although it is inefficient, one-step enzymatic hydrolysis is environmental-friendly. Thus, it is a good strategy to research the synergistic effects of enzymes and to improve the binding ability of enzymes for insoluble substrates.

Carbohydrate-binding modules (CBMs) exist in many glycoside hydrolases including xylanases, and are independent and non-catalytic areas of the structure (Antoine et al. 2004; Rodriguez-Sanoja et al. 2009). Because of its significant role in improving the catalytic efficiency of enzymes, many researchers have studied and reported on this module. While comparing the kinetic constants of CBM1, CBM2, CBM3, CBM4, CBM10, CBM72 in cellulase Umcel9A and CBM-chimeric Umcel9A, the *k*_cat_/*K*_m_ values of the chimeric enzymes were 1.09–4.44 times more than those of the wild-type (Duan et al. 2017). Furthermore, the catalytic efficiency of acetyl xylan esterase AnAXE–*Ct*CBM3 was approximately 5% more than that of wild-type AnAXE towards *p*NPA (Mai-Gisondi et al. 2015). While comparing of the acetyl xylan esterase properties of AXE + CBM1 and AXE from *Talaromyces leycettanus* JCM12802, the optimum conditions were found to be uniform and the pH stability was equally broad as well. This result indicated that the elimination of the CBM1 structural domain had no effect on the basic enzymatic properties of AXE. To study xylanase, *NP*XYN11 (GenBank: AF123252.1) from *Neocallimastix patriciarum* and belonging to the GH11 family was used. Because of its excellent thermal stability and high specific activity, many scholars, such as Bu et al. (2018), Chen et al. (2001), Malunga and Beta (2015), and Krause et al. (2001), have conducted detailed studies with respect to this xylanase in recent years. Although it has satisfactory industrial application prospects, *NP*XYN11 has not a CBM domain and therefore, the CBM1 domain was introduced at the C-terminal of the protein. Upon comparing the basic nature of *NP*XYN11 and *NP*XYN11 + CBM1, their temperature and pH of optimum and stability were found to basically be the same. This means that the addition of an exogenous CBM1 domain did not affect the basic properties of *NP*XYN11. However, the deletion and addition of the CBM1 domain was found to influence kinetic parameters (Table 2). When the *k*_cat_/*K*_m_ values for AXE + CBM1 and *NP*XYN11 + CBM1 were compared with those of AXE and *NP*XYN11 respectively, a rising trend of approximately 25%–35% was observed.

Furthermore, acetylation was shown to limit the degradation of acylated xylanase (Selig et al. 2009); however, the addition of acetyl xylan esterase solved this problem. The synergistic reaction was reflected as follows: acetyl xylan esterase helped xylanase to degrade xylan in order to produce more reducing sugars, and xylanase assisted acetyl xylan esterase in removing acetylates in order to generate acetic acid. The α-l-arabinofuranosidase is an assistant enzyme responsible for xylan depolymerization, and is widely studied because it can assist xylanase during reactions (Goncalves et al. 2012). The amounts of reducing sugars produced by α-l-arabinofuranosidase and xylanase from oat-spelt xylan were confirmed to be greater by about approximately 30% when compared with the amount from single xylanase (Huang et al. 2017). The outstanding synergistic effects of α-l-arabinofuranosidase Ac-Abf51A and xylanase XynBE18 was observed to increase by 2.92 folds during water-soluble wheat arabinoxylan degradation (Yang et al. 2015a). However, only a few studies exist on the synergistic effects of acetyl xylan esterase and xylanase, especially with respect to wheat bran as a natural substrate. After previous exploration, 200 U of xylanase 50 U of acetyl xylan esterase were found to exert the maximum effect (Fig. 4). Moreover, the addition of acetyl xylan esterase and xylanase, in that order, produced the largest amount of reducing sugars (Table 3). The synergistic effect was also observed to be the best when the auxiliary enzyme first reacted with the substrate and then added to the main chain enzyme. This was consistent with the 1.2-fold increase found in the study showed the synergistic effect of xylanase and α-l-arabinofuranosidase on birchwood xylan (Yang et al. 2015b). When AXE + CBM1 or AXE was added first, the respective reducing sugar content were 29% or 16% higher than that produced when only xylanase was reacted. Since side chain groups are sterically hindered by the degradation of main chain enzymes, acetyl groups may affect the degradation of xylan structures via xylanase. Therefore, the removal of side chain substituent via side chain enzyme is more conducive to the degradation activity of the main chain enzyme. It was speculated that acetyl xylan esterase excised the acetyl group on the side chain of xylanase, which allowed xylanase to move closer to the main chain of xylan. Notably, the effect of xylanase with the CBM1 domain was more obvious at higher substrate concentrations. The amount of reducing sugars were also observed to be slightly higher when AXE or AXE + CBM1 were added later when compared to xylanase, the content of reducing sugar contents were 4% or 6% higher than that produced when only xylanase was added. The reason for this phenomenon may be because the main chain degradation of xylan by xylanase generates different sizes of fragments, then acetyl xylan esterase effects on these fragments, resulting in a small amount of reduction and thus, so a small increase reducing sugars increased less (Zhu et al. 2016). When all conditions were optimal, *NP*XYN11 + CBM1 and AXE + CBM1 produced 34% more reducing sugars than *NP*XYN11 and AXE independently.

Both xylobiose and xylotriose are basic components of xylooligosaccharides. Interestingly higher are the activity and purity of xylooligosaccharide products are correlated with higher xylobiose and xylotriose contents (Antoine et al. 2004). In addition, xylobiose is an alternative healthy sweetener that helps avoid obesity. A study by Soo-min Lim showed that adding 10% of xylobiose to mouse diets effectively suppressed fat accumulation in the mesenteric (29%), subcutaneous (22%), and perirenal (16%) deposits (Lim et al. 2018). Furthermore, the expressed recombinant xylanase Xyn2 in *P. pastoris* and its products from hydrolyzed oat-spelt xylan were mostly xylotriose, and supplementation with 500 U kg^−1^ of Xyn2 on average produced a daily body-weight gain of 16.9% in weaned pigs (He et al. 2009). In this study, the results showed that when wheat bran was used as a substrate, not only did acetyl xylan esterase and xylanase together with the CBM1 domain produce nearly 35% more reducing sugars than enzyme reactions without the CBM1 domain, but the introduction of xylanase into the CBM1 domain also did not change the composition of xylose, the highest proportion of which was 86%, that consisted of 49% xylobiose and 37% xylotriose.

## Abbreviations

CBM: carbohydrate-binding module
GH: glycoside hydrolase
AXE + CBM1: a full-length acetyl xylan esterase from *Talaromyces leycettanus* JCM12802
AXE: the CBM1 and Linker domain removed AXE + CBM1
*NP*XYN11: a GH11 family xylanase from *Neocallimastix patriciarum*
*NP*XYN11 + CBM1: the CBM1 and Linker domain added *NP*XYN11

## References

[CR1] Antoine C, Peyron S, Lullien-Pellerin V, Abecassis J, Rouau X (2004). Wheat bran tissue fractionation using biochemical markers. J Cereal Sci.

[CR2] Bastawde KB (1992). Xylan structure, microbial xylanases, and their mode of action. World J Microbiol Biotechnol.

[CR3] Biely P, Mislovicova D, Toman R (1985). Soluble chromogenic substrates for the assay of endo-1,4-beta-xylanases and endo-1,4-beta-glucanases. Anal Biochem.

[CR4] Biely P, Vrsanska M, Tenkanen M, Kluepfel D (1997). Endo-beta-1,4-xylanase families: differences in catalytic properties. J Biotechnol.

[CR5] Bu Y, Cui Y, Peng Y, Hu M, Tian Y, Tao Y, Wu B (2018). Engineering improved thermostability of the GH11 xylanase from *Neocallimastix patriciarum* via computational library design. Appl Microbiol Biotechnol.

[CR6] Chakdar H, Kumar M, Pandiyan K, Singh A, Nanjappan K, Kashyap PL, Srivastava AK (2016). Bacterial xylanases: biology to biotechnology. 3 Biotech.

[CR7] Chen YL, Tang TY, Cheng KJ (2001). Directed evolution to produce an alkalophilic variant from a *Neocallimastix patriciarum* xylanase. Can J Microbiol.

[CR8] Chen LL, Zhang M, Zhang DH, Chen XL, Sun CY, Zhou BC, Zhang YZ (2009). Purification and enzymatic characterization of two beta-endoxylanases from *Trichoderma* sp. K9301 and their actions in xylooligosaccharide production. Bioresour Technol..

[CR9] Collins T, Gerday C, Feller G (2005). Xylanases, xylanase families and extremophilic xylanases. FEMS Microbiol Rev.

[CR10] Cybinski DH, Layton I, Lowry JB, Dalrymple BP (1999). An acetylxylan esterase and a xylanase expressed from genes cloned from the ruminal fungus *Neocallimastix patriciarum* act synergistically to degrade acetylated xylans. Appl Microbiol Biotechnol.

[CR11] Duan CJ, Huang MY, Pang H, Zhao J, Wu CX, Feng JX (2017). Characterization of a novel theme C glycoside hydrolase family 9 cellulase and its CBM-chimeric enzymes. Appl Microbiol Biotechnol.

[CR12] Goncalves TA, Damasio AR, Segato F, Alvarez TM, Bragatto J, Brenelli LB, Citadini AP, Murakami MT, Ruller R, Paes Leme AF, Prade RA, Squina FM (2012). Functional characterization and synergic action of fungal xylanase and arabinofuranosidase for production of xylooligosaccharides. Bioresour Technol.

[CR13] He J, Yu B, Zhang KY, Chen DW (2009). Functional characterization of a recombinant thermostable xylanase from *Pichia pastoris*: a hybrid enzyme being suitable for xylooligosaccharides production. Biochem Eng J.

[CR14] Ho AL, Carvalheiro F, Duarte LC, Roseiro LB, Charalampopoulos D, Rastali RA (2014). Production and purification of xylooligosaccharides from oil palm empty fruit bunch fibre by a non-isothermal process. Bioresour Technol.

[CR15] Hsu CK, Liao JW, Chung YC, Hsieh CP, Chan YC (2004). Xylooligosaccharides and fructooligosaccharides affect the intestinal microbiota and precancerous colonic lesion development in rats. J Nutr.

[CR16] Huang D, Liu J, Qi YF, Yang KX, Xu YY, Feng L (2017). Synergistic hydrolysis of xylan using novel xylanases, beta-xylosidases, and an alpha-l-arabinofuranosidase from *Geobacillus thermodenitrificans* NG80-2. Appl Microbiol Biotechnol.

[CR17] Krause DO, Bunch RJ, Dalrymple BD, Gobius KS, Smith WJ, Xue GP, McSweeney CS (2001). Expression of a modified *Neocallimastix patriciarum* xylanase in *Butyrivibrio fibrisolvens* digests more fibre but cannot effectively compete with highly fibrolytic bacteria in the rumen. J Appl Microbiol.

[CR18] Lim SM, Kim E, Shin JH, Seok PR, Jung S, Yoo SH, Kim Y (2018). Xylobiose prevents high-fat diet induced mice obesity by suppressing mesenteric fat deposition and metabolic dysregulation. Molecules.

[CR19] Maes C, Delcour JA (2002). Structural characterisation of water-extractable and water-unextractable arabinoxylans in wheat bran. J Cereal Sci.

[CR20] Mahadevan SA, Wi SG, Kim YO, Lee KH, Bae HJ (2011). In planta differential targeting analysis of *Thermotoga maritima* Cel5A and CBM6-engineered Cel5A for autohydrolysis. Transgenic Res.

[CR21] Mai-Gisondi G, Turunen O, Pastinen O, Pahimanolis N, Master ER (2015). Enhancement of acetyl xylan esterase activity on cellulose acetate through fusion to a family 3 cellulose binding module. Enzyme Microb Technol.

[CR22] Malunga LN, Beta T (2015). Antioxidant capacity of arabinoxylan oligosaccharide fractions prepared from wheat aleurone using Trichoderma viride or *Neocallimastix patriciarum* xylanase. Food Chem.

[CR23] Matsui I, Ishikawa K, Matsui E, Miyairi S, Fukui S, Honda K (1991). Subsite structure of Saccharomycopsis alpha-amylase secreted from Saccharomyces cerevisiae. J Biochem.

[CR24] Mcdermid KP, Forsberg CW, MacKenzie CR (1990). Purification and properties of an acetyl xylan esterase from *Fibrobacter succinogenes* S85. Appl Environ Microbiol.

[CR25] Miao Y, Li P, Li G, Liu D, Druzhinina IS, Kubicek CP, Shen Q, Zhang R (2017). Two degradation strategies for overcoming the recalcitrance of natural lignocellulosic xylan by polysaccharides-binding GH10 and GH11 xylanases of filamentous fungi. Environ Microbiol.

[CR26] Peralta AG, Venkatachalam S, Stone SC, Pattathil S (2017). Xylan epitope profiling: an enhanced approach to study organ development-dependent changes in xylan structure, biosynthesis, and deposition in plant cell walls. Biotechnol Biofuels.

[CR42] Puls J, Tenkanen M, Korte HE, Poutanen K (1991). Products of hydrolysis of beechwood acetyl-4-O-methylglucuronoxylan by a xylanase and an acetyl xylan esterase. Enzyme Microb Technol.

[CR27] Qi BK, Chen XR, Shen F, Su Y, Wan YH (2009). Optimization of enzymatic hydrolysis of wheat straw pretreated by alkaline peroxide using response surface methodology. Ind Eng Chem Res.

[CR28] Quinones TS, Retter A, Hobbs PJ, Budde J, Heiermann M, Plochl M, Ravella SR (2015). Production of xylooligosaccharides from renewable agricultural lignocellulose biomass. Biofuels.

[CR29] Rastall RA (2010). Functional oligosaccharides: application and manufacture. Annu Rev Food Sci Technol.

[CR30] Reddy SS, Krishnan C (2016). Production of high-pure xylooligosaccharides from sugarcane bagasse using crude beta-xylosidase-free xylanase of *Bacillus subtilis* KCX006 and their bifidogenic function. Lwt Food Sci Technol.

[CR31] Rodriguez-Sanoja R, Oviedo N, Escalante L, Ruiz B, Sanchez S (2009). A single residue mutation abolishes attachment of the CBM26 starch-binding domain from *Lactobacillus amylovorus* alpha-amylase. J Ind Microbiol Biotechnol.

[CR32] Selig MJ, Adney WS, Himmel ME, Decker SR (2009). The impact of cell wall acetylation on corn stover hydrolysis by cellulolytic and xylanolytic enzymes. Cellulose.

[CR33] Shimizu K, Sudo K, Ono H, Ishihara M, Fujii T, Hishiyama S (1998). Integrated process for total utilization of wood components by steam-explosion pretreatment. Biomass Bioenergy.

[CR34] Strelova O, Chuvina NA (2008). Isolation of blood caffeine as a model substance by enzymatic hydrolysis. Sud Med Ekspert.

[CR35] Sun Z, Lv W, Yu R, Yu J, Liu H, Sun W, Wang Z, Li J, Shan Z, Qin Y (2013). Effect of a straw-derived xylooligosaccharide on broiler growth performance, endocrine metabolism, and immune response. Can J Vet Res.

[CR36] Vazquez MJ, Alonso JL, Dominguez H, Parajo JC (2000). Xylooligosaccharides: manufacture and applications. Trends Food Sci Technol.

[CR37] Wang S, Zhang P, Kong X, Xie S, Li Q, Li Z, Zhou Z (2017). Delicate changes of bioapatite mineral in pig femur with addition of dietary xylooligosaccharide: evidences from raman spectroscopy and ICP. Animal Sci J.

[CR38] Yang W, Bai Y, Yang P, Luo H, Huang H, Meng K, Shi P, Wang Y, Yao B (2015). A novel bifunctional GH51 exo-alpha-l-arabinofuranosidase/endo-xylanase from *Alicyclobacillus* sp. A4 with significant biomass-degrading capacity. Biotechnol Biofuels.

[CR39] Yang X, Shi P, Ma R, Luo H, Huang H, Yang P, Yao B (2015). A new GH43 alpha-arabinofuranosidase from Humicola insolens Y1: biochemical characterization and synergistic action with a xylanase on xylan degradation. Appl Biochem Biotechnol.

[CR40] Yang J, Li Q, Henning SM, Zhong J, Hsu M, Lee R, Long J, Chan B, Nagami GT, Heber D, Li Z (2018). Effects of prebiotic fber xylooligosaccharide in adenine-induced nephropathy in mice. Mol Nutr Food Res.

[CR41] Zhu T, Yao HE, Yang B, Liu J, Minchen W (2016). Combinative degradation of xylan with acetyl xylan esterase and xylanase. J Food Sci Biotechnol.

